# Women’s Brain Health: Midlife Ovarian Removal Affects Associative Memory

**DOI:** 10.1007/s12035-023-03424-6

**Published:** 2023-07-10

**Authors:** Alana Brown, Nicole J. Gervais, Jenny Rieck, Anne Almey, Laura Gravelsins, Rebekah Reuben, Laurice Karkaby, M. Natasha Rajah, Cheryl Grady, Gillian Einstein

**Affiliations:** 1 Psychology, University of Toronto, 100 St. George Street, Toronto, ON M5S 3G3 Canada; 2 Rotman Research Institute, Baycrest Health Sciences, Toronto, M6A 2E1 Canada; 3 Departments of Psychiatry and Douglas Research Centre, McGill University, Montreal, H4H 1R3 Canada; 4 Psychiatry, University of Toronto, Toronto, M5T 1R8 Canada; 5 Linköping University, 581 83 Linköping, Sweden

**Keywords:** Menopause, Oophorectomy, Associative memory, Hippocampus, Estradiol, Functional magnetic resonance imaging

## Abstract

Women with early bilateral salpingo-oophorectomy (BSO; removal of ovaries and fallopian tubes) have greater Alzheimer’s disease (AD) risk than women in spontaneous/natural menopause (SM), but early biomarkers of this risk are not well-characterized. Considering associative memory deficits may presage preclinical AD, we wondered if one of the earliest changes might be in associative memory and whether younger women with BSO had changes similar to those observed in SM. Women with BSO (with and without 17β-estradiol replacement therapy (ERT)), their age-matched premenopausal controls (AMC), and older women in SM completed a functional magnetic resonance imaging face-name associative memory task shown to predict early AD. Brain activation during encoding was compared between groups: AMC (*n*=25), BSO no ERT (BSO; *n*=15), BSO+ERT (*n*=16), and SM without hormone therapy (*n*=16). Region-of-interest analyses revealed AMC did not contribute to functional group differences. BSO+ERT had higher hippocampal activation than BSO and SM. This hippocampal activation correlated positively with urinary metabolite levels of 17β-estradiol. Multivariate partial least squares analyses showed BSO+ERT had a different network-level activation pattern than BSO and SM. Thus, despite being approximately 10 years younger, women with BSO without ERT had similar brain function to those with SM, suggesting early 17β-estradiol loss may lead to an altered functional brain phenotype which could influence late-life AD risk, making face-name encoding a potential biomarker for midlife women with increased AD risk. Despite similarities in activation, BSO and SM groups showed opposite within-hippocampus connectivity, suggesting menopause type is an important consideration when assessing brain function.

## Introduction

Two thirds of Alzheimer disease (AD) sufferers are women, and women with early bilateral salpingo-oophorectomy (BSO; removal of ovaries and fallopian tubes) prior to spontaneous/natural menopause (SM) have greater risk of late-life AD and accelerated cognitive decline compared to women in SM [1–3]. Thus, it is important to understand how this particular risk factor influences women’s progression to AD and whether markers of this risk can be detected in midlife.

Functional magnetic resonance imaging (fMRI) has been a mainstay of efforts to develop early markers of late-life AD. Cross-modal associative memory tasks, such as face-name paradigms, are sensitive to genetic AD risk (apolipoprotein ε4; APOE4), amyloid-β burden, and mild cognitive impairment progression [4–8]. Associative memory-related brain network dysfunction occurs prior to amyloid-β accumulation [9], suggesting that functional alterations are among the most sensitive means for assessing early AD changes. Face-name pair encoding requires activation of a distributed set of brain regions, including frontoparietal cortex and hippocampus [8, 10]. The effects of age and AD on patterns of activity during associative encoding in these regions include decreased activation [8, 11]. Although evidence suggests associative task performance and brain activation depend on women’s reproductive stage and are linked to fluctuating 17β-estradiol levels, most studies come from older and mixed-sex cohorts that do not consider potential midlife AD risk factors for women, such as BSO [12, 13].

Considerable data indicate 17β-estradiol and progesterone affect synaptic function and organization within the frontal cortex and hippocampus [12, 14–18], and their receptors are abundant throughout these brain areas [15, 19]. In rodents, ovarian removal leads to decreased dendritic spine density in hippocampal Cornu Ammonis 1 pyramidal neurons, which is prevented by 17β-estradiol administration [20]. Further, 17β-estradiol plays a role in protecting rodent hippocampal neurons from amyloid beta-induced apoptosis via regulation of mitochondrial proteins and function [21]. In humans, during associative learning, hippocampal activation declines and bilateral hippocampal connectivity increases across the menopause transition [12]. Decreased hippocampal volume and function in SM may be related to 17β-estradiol loss, which is supported by work showing the loss may be ameliorated by 17β-estradiol replacement therapy (ERT) [22–24]. ERT also preserves dorsolateral prefrontal cortical volume in SM [25]. Recent research has further linked midlife BSO and its concomitant 17β-estradiol loss with thinner parahippocampal-entorhinal cortices later in life and reduced hippocampal volume within 5 years of BSO [26, 27].

Perhaps due to these brain alterations, 17β-estradiol loss in SM is associated with decreased episodic verbal memory and associative memory compared to pre- and peri-menopausal women [13]. Women with bilateral oophorectomy have some of the same memory reductions but 10 years earlier, and evidence suggests ERT may maintain verbal episodic memory and working memory among women in SM and with oophorectomy [28–32]. Cumulatively, these findings suggest ovarian hormone loss in any type of menopause can negatively affect memory, but changes may occur earlier in women with midlife BSO. Thus, determining memory changes in women with BSO may provide insight into the earliest brain changes presaging AD.

Given that associative memory decline is a hallmark of AD, our objective was to determine if associative memory changes are present in women with midlife BSO. Further, we wished to determine whether changes in women with BSO resemble those of women in SM, only approximately 10 years earlier. To do this, we carried out a cross-sectional fMRI study comparing women with early BSO to age-matched premenopausal controls with their ovaries (AMC) and older women in SM on an associative task known to reveal some of the earliest functional brain changes related to AD [8]. We wondered whether associative memory would be worse in women with BSO compared to AMC and women in SM, and whether ERT in women with BSO would preserve performance and/or encoding-related function. To answer these questions, we determined whether performance on a face-name associative memory task and brain function and connectivity during the encoding phase of this task differed between women with BSO taking and not taking ERT, AMC, and SM groups.

## Materials and Methods

### Recruitment

This study was performed in line with the principles of the Declaration of Helsinki. Approval was granted by the Ethics Committees of the University of Toronto and McGill University. Informed consent was obtained from all individual participants included in the study.

Women in this study were recruited from a larger cohort [32]. Exclusion criteria for all participant groups included non-fluency in English, contraindications for MRI safety, perimenopause, BSO after SM, past hormone therapy use (BSO and SM), unmanaged health/psychiatric conditions, history of concussion with loss of consciousness, or chemo/radiation/adjuvant therapies within 6 months of testing. All women with cancer treatment history were 6 months or more post-treatment, with an average testing date at 7 years post-treatment. Nonetheless, we included cancer treatment history as a covariate in all univariate analyses.

Women with BSO were excluded if they were using a non-ERT form of hormone therapy (e.g., conjugated equine estrogen); AMC and SM participants if they were using hormone therapy or hormonal contraceptives. Women in the AMC group had experienced regular menstrual cycling within 6 months prior to the study date. Women in the SM group had their last menstrual period 10 or more months prior to study onset; removing the three participants with their last menstrual period between 10 and 12 months prior to the study date did not alter region-of-interest (ROI) or accuracy results.

A total of 72 women were included in the study and were separated into four groups: (1) women with BSO not taking hormone therapy (BSO), (2) women with BSO taking ERT (BSO+ERT), (3) premenopausal control women with their ovaries, age-matched to women with BSO (AMC), and (4) women in SM not taking hormone therapy (SM) (Table 1). Participants with BSO were recruited from familial breast and ovarian cancer clinics in Toronto and Montreal, Canada; AMC and SM participants were recruited from the general community in the same cities.

**Table 1:** Demographic and behavioral characteristics

	Total *(n*=72; age range, 33–59)		BSO combined (combined groups not taking hormone therapy and taking ERT; *n*=31; age range, 35–55)						AMC (*n*=25, age range, 33–51)		SM (*n*=16; age range, 47–59)	
					BSO *(n*=15; age range, 35–55)		BSO+ERT *(n*=16; age range, 38–55)					
Characteristic	Mean	SEM	Mean	SEM	Mean	SEM	Mean	SEM	Mean	SEM	Mean	SEM
Age (years)	47.03	0.81	45.67	1.03	46.53	1.51	44.75	1.33	43	0.91	56.06^a^	0.81
Education (years)	17.87	0.35	17.96	0.51	18.21	0.60	17.53	0.79	18.54	0.73	16.88	0.54
BMI (kg/m^2^)	25.02	0.51	25.58	0.86	25.65	0.81	25.31	1.46	24.29	0.86	25.20	0.91
Verbal IQ	39.49	1.31	38.68	2.24	40.07	2.95	37.27	3.20	40.10	1.80	40.31	2.94
CES-D	9.51	0.94	9.77	1.65	8.6	1.99	11.19	2.50	10.40	1.55	7.31	1.51
PSS	14.78	0.85	14.70	1.36	14.53	1.84	15.06	1.94	16.60	1.41	11.88	1.61
Age at menopause (years)	44.38	0.97	41.10	0.89	42	1.42	39.81	1.06	NA	NA	51.19^a^	0.82
Time since menopause (years)	4.79	0.49	4.55	0.55	4.59	0.77	4.87	0.84	NA	NA	4.89	0.98
Urinary E1G (ng/ml)	28.79	2.40	28	3.44	19.85	2.13	34.74^b^	5.70	37.46	4.74	17.68	3.30
Urinary PdG (μ/ml)	4.83	1.41	7.99	3.28	2.28	1.12	13.19	6.03	4.01	1.00	0.67^b^	0.15
Face-name task accuracy (%)	83.88	1.32	84.64	1.95	85.71	2.49	83.93	2.88	87.43^a^	2.14	76.56	2.61
	*n*	%	*n*	%	*n*	%	*n*	%	*n*	*%*	*n*	%
Current smoker	5	6.94	0	0	0	0	1	6.25	2	8	2	12.5
APOE4 genotype	13	18.06	4	12.90	4	26.67	0	0	7	28	2	13.33
Right-handedness	66	91.67	27	87.10	14	93.33	13	81.25	23	92	16	100
History of chemotherapy	8	11.11	7	22.58	6^c^	40	1	6.25	0	0	1	6.25
History of other cancer treatment	7	9.72	6	19.35	6^c^	40	0	0	0	0	1	6.25
Total history of cancer treatment	10	13.89	8	25.81	7^c^	46.67	1	6.25	0	0	2	12.5

History of other cancer treatment included radio-therapy, and/or adjuvant tamoxifen use

Post hoc comparisons: Age: SM>AMC=BSO=BSO+ERT; Age at menopause: SM>BSO=BSO+ERT; Urinary E1G: BSO+ERT=AMC>BSO=SM; PdG: SM<AMC=BSO+ERT, AMC=BSO=BSO+ERT, BSO=SM; Face-name task accuracy: AMC>SM; History of cancer treatment: BSO>AMC=BSO+ERT=SM

Verbal IQ was estimated from the North American Adult Reading Test

Abbreviations: SEM standard error of the mean, *SM* spontaneous menopause, *BSO* bilateral salpingo-oophorectomy, *BSO+ERT* bilateral salpingo-oophorectomy with 17β-estradiol replacement therapy, with or without other hormone therapy types, *BMI* body mass index, *APOE4* apolipoprotein E4 allele (genetic risk factor for AD), *CES-D* Center for Epidemiological Studies—Depression Scale, *PSS* Perceived Stress Scale, *E1G* estrone-3-glucuronide, *PdG* pregnanediol glucuronide, *NA* not applicable

^a^Significant (*p*<0.05) post hoc Tukey’s HSD test

^b^Significant Dunn test

^c^Significant Pearson’s Chi-squared test

### Image Acquisition 

In Toronto, images were acquired on Siemens 3T MAGNETOM Prisma scanners at Baycrest Health Sciences Centre and the Toronto Neuroimaging Institute at the University of Toronto. In Montreal, images were acquired on a 3T MAGNETOM Prisma-Fit scanner at the Douglas Hospital Research Institute Brain Imaging Center. Functional images were acquired with a T2*-weighted blood-oxygen-level-dependent gradient echo-planar imaging sequence with 39 coronal, interleaved slices perpendicular to the anterior/posterior commissure line, with voxel dimensions=3.5mm^3^ with 1-mm interslice gap, field of view (FOV)=224mm^2^, flip angle=70°, echo time (TE)=27ms, repetition time (TR)=2000ms, and image dimensions=64×64×39mm. T1-weighted anatomical images were acquired using a 3D gradient echo MPRAGE sequence with voxel dimensions=1.0mm^3^, 160 sagittal slices, FOV=256mm^2^, TE=2.67ms, TR=2000ms, and flip angle=9°.

### Image Preprocessing

Following image acquisition and prior to statistical analysis, fMRI data were converted to NIfTI format and preprocessed using a standard pipeline implemented through OPPNI (Optimizing of Preprocessing Pipelines for NeuroImaging), a software package that uses functions developed by researchers at the Rotman Research Institute [33], Analysis of Functional NeuroImages (AFNI [34]), and FMRIB Software Library (FSL [35]), to control for sources of noise and artefact. For the current study, a standard conservative pipeline was chosen in which the same preprocessing steps were applied to all groups and conditions.

FMRI images were adjusted to the anterior/posterior commissure plane. They were corrected for head motion using AFNI’s 3dvolreg with MOTCOR, which aligned each volume to a reference volume, estimated as being the least affected by head motion. Consequently, values from volumes not matching the reference volume were substituted with values from neighboring matching volumes. Removal of estimated physiological noise components was accomplished using PHYPLUS, an Octave script developed in-house that utilizes the data driven PHYCAA+ algorithm to identify and remove noise with a strong vascular component.

Using AFNI’s 3dmerge with SMOOTH, images were then spatially smoothed by 6mm, convolving blood-oxygen-level-dependent (BOLD) signal with a 3D isotropic full-width-at-half-maximum Gaussian kernel to reduce signal noise and ameliorate differences in inter-subject localization. Each participant also had a non-neuronal tissue mask created to discard confounding signal coming from non-brain tissue (ventricles, vasculature, and sinuses). Temporal detrending was applied using DETREND, which used a quadratic polynomial function (maximum polynomial order 2) fitted to regress out low frequency noise. Further motion correction for residual artefacts was also completed. Finally, task design was inputted in the model and mapped onto the hemodynamic response function to ensure the noise regressed out in previous stages was not related to task activation.

Two final steps not belonging to the OPPNI pipeline were conducted: spatial normalization both in the participant’s native space and in standard space, as each participant’s functional images were co-registered to their T1 structural image and normalized and registered to 4mm^3^ standard space using FSL’s anatomical 2mm^3^ MNI152 brain template. Noise from white matter, cerebrospinal fluid, and vessels was also removed due to potential interference with the BOLD signal of interest from grey matter.

### Procedure

Face stimuli [36] were presented using E-Prime [37] on a black background with an English name printed underneath each face followed by a white fixation cross on a black background. An event-related design was used. Participants were scanned during the first-time encoding of 30 face-name pairs (including 28 novel face-name pairs and two subsequently repeated face-name pairs). Each novel face-name pair (for the Novel condition) was presented once for 4.5 s. Two repeated face-name pairs (for the Repeat condition) were presented for 4.5 s each, with the face-name pairs alternating throughout each repeated block. Each session had a total duration of 8.75 min for 278 scans and was composed of four Novel blocks alternating with four Repeat blocks. Each block comprised seven face-name pairs. As each face-name pair appeared on the screen, participants indicated whether they thought the presented name was a “good” or “bad” name for the face by pressing buttons on a response pad.

Approximately 30 min later, outside of the scanner, participants performed a recognition task on a PC computer in which they chose the name (from two alternatives) that was previously paired with each face they saw during the scans. This recognition phase comprised 27 face-name pairs that were seen during the Novel condition, and one pair that was seen during the Repeat condition in the scanner, for a total of 28 test trials. Participants provided a urine sample to assess levels of metabolites of 17β-estradiol (estrone-3-glucuronide; E1G) and progesterone (pregnanediol-3 glucuronide; PdG). Urine samples were analyzed at the Women’s Health and Exercise Laboratory at Pennsylvania State University using enzyme-linked immunoassays described by Munro and colleagues [38]. Apolipoprotein E genotype was determined using a saliva sample processed at The Hospital for Sick Children in Toronto.

### Statistical Analysis

#### Univariate Analysis

We first wanted to determine whether there was a general effect of BSO on task performance and ROI activation. Thus, we first assessed differences between BSO (combined taking ERT and not taking ERT), AMC, and SM groups. We then wanted to assess the effect of ERT on task performance and ROI activation; therefore, we separated the combined BSO group into BSO (no hormone therapy) and BSO+ERT, and compared these groups to AMC and SM groups.

SPM12 (Statistical Parametric Mapping; Wellcome Trust Centre for Neuroimaging) was used to analyze the functional data. Mean activity for each image voxel was modeled independently for the task conditions (Novel/Repeat). To model BOLD response to experimental conditions separately, a mixed-effects general linear model was fit for every participant at the individual level.

The fMRI general linear model first-level analysis consisted of two explanatory variables: (1) encoding of novel face-name pairs (Novel condition), and (2) encoding of repeated face-name pairs (Repeat condition). Each of these was convolved with a hemodynamic response function and temporally filtered with a high-pass cutoff of 260 s to remove low-frequency signal drifts [8]. The primary first-level contrast of interest was Novel–Repeat.

**Fig. 1:**
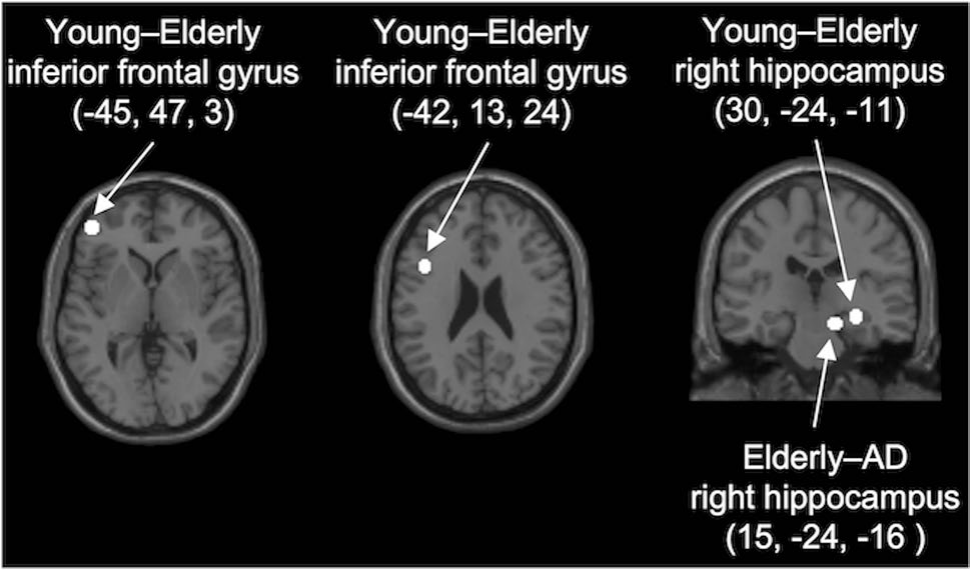
Locations of spherical regions-of-interest from coordinates from Sperling and colleagues [8] are shown in Montreal Neurological Institute (MNI) space

ROIs were generated by positioning the center of a 6-mm diameter sphere around coordinates defined a priori showing significantly higher activation in mixed-sex groups of young versus elderly adults (Young–Elderly contrast) as well as in mixed-sex groups of elderly adults versus patients with AD (Elderly–AD contrast) [8]. Due to demonstrated involvement in functional brain aging and AD, we explored a total of four ROIs, all of them in frontal or hippocampal cortices. We included three ROIs from the Young–Elderly contrast coordinates, consisting of two left inferior frontal gyrus ROIs (centered upon the following coordinates: *X*=−45, *Y*=47, *Z*=3; *X*=−42, *Y*=13, *Z*=24), and one ROI in the right hippocampus (centered upon the following coordinates: *X*=30, *Y*=−24, *Z*=−11). A second ROI in the right hippocampus was included from the Elderly–AD contrast (centered upon the following coordinates: *X*=15, *Y*=−24, *Z*=−16). Hippocampal ROIs (Young–Elderly and Elderly–AD) were located in the right medial and lateral regions of the posterior/mid hippocampus [39]. The ROI centered upon coordinates from the Young–Elderly contrast was more lateral/proximal than the ROI centered upon coordinates from the Elderly–AD contrast (Fig. 1).

Parameter estimates were extracted from each ROI (averaged across all voxels in each ROI) using MarsBaR [40]. ROI analyses were performed in R 4.0.2 (R Core Team, 2019). ROI parameter estimates (activation) and face-name associative task accuracy were predicted using a one-way analysis of covariance (ANCOVA) with Tukey’s post hoc tests and group as a between-subjects factor, controlling for scanner site [41] for parameter estimate analyses and history of cancer treatment for both parameter estimate and associative task accuracy analyses. Additionally, exploratory Spearman correlations between ovarian hormone levels, face-name task performance, and mean ROI parameter estimates were run.

Age and hormone deprivation are interacting factors; therefore, age was not included as a covariate in these analyses, as removal of variance accounted for by age could also involve removal of variance shared with hormone deprivation. As appropriate, ANCOVAs or Kruskal-Wallis tests were conducted, where ROI relationships were considered significant only if they met the Bonferroni correction for multiple comparisons of *p*<0.013 (*p*<0.05/4 ROIs). ANOVA, Kruskal-Wallis, or Chi-squared tests were conducted to compare groups on demographic variables. Due to skewness, extreme E1G and PdG level values were Winsorized to the value at the 90th or 10th percentile of the distribution. Post hoc comparisons were conducted using Tukey’s HSD or Benjamini-Hochberg adjustment tests, as appropriate. G*Power version 3.1.9.4 was used to calculate post hoc power for examining univariate group differences.

#### Exploratory Partial Least Squares (PLS) Analysis

The AMC group did not contribute to significant group ROI activation differences; therefore, we decided to run exploratory whole-brain multivariate partial least squares (PLS; McIntosh and Lobaugh,[42]

)analyses among BSO, BSO+ERT, and SM groups to further investigate differences in Novel and Repeat conditions across distributed patterns of activated voxels throughout the brain. One major advantage of multivariate Task PLS is that multiple comparisons across brain regions do not need to be corrected for in whole-brain function assessment [42. ]. Unlike univariate analyses, PLS does not require meeting assumptions of normality, independence of observations, and linearity for general linear models [43]. Therefore, it is possible to look at many brain regions simultaneously. PLS calculates a covariance matrix between brain voxels and experimental design across participants to identify a new set of variables (latent variables or LVs) that optimally explain covariance between conditions and brain activity.

Each LV contains a spatial activity pattern of brain regions that, together, show the strongest relation to the contrast of conditions and groups identified by the LV. The covariance matrix undergoes singular value decomposition (SVD), a data reduction tool. As SVD is completed in one step, no correction for multiple comparisons is needed, increasing sensitivity and robustness compared to univariate analysis [44]. Data were mean-centered by subtracting condition and group means, and brain scores were calculated for each condition.

The statistical significance of each LV was determined by conducting 1000 permutation tests on the singular values. To assess the reliability of each voxel’s contribution to an LV, 1000 bootstrap samples of saliences were conducted. Confidence intervals (95%) for the mean brain scores in each condition and group were calculated from the bootstrap, and differences in activity between conditions and groups were determined via lack of overlap in these confidence intervals. Local maxima in the brain were considered reliable if the bootstrap ratio (BSR; computed as the ratio of a voxel’s salience to the bootstrap standard error) for the regions was ±2.50 (*p* < 0.012) with a minimum cluster size of 10 voxels and a minimum cluster distance of 10mm. Voxels were labeled using the Automatic Anatomical Labeling atlas [45]. The Hemodynamic Response Function (HRF) for a given condition typically lasts for several scans; thus, a “lag-window” representing the response of each voxel with each trial is determined. Based on findings suggesting that hippocampal activity is particularly enhanced at the end of an event [46], we focused on local maxima for the 6th and 7th lags. Given that a sample size of approximately 80 or more participants would be needed for stable estimates of brain-behavior correlation magnitudes [47], we decided not to run behavioral PLS analyses.

#### Exploratory Connectivity PLS Analysis

Using the CONN toolbox, we assessed whether functional connectivity between the left and right anterior and posterior hippocampi differed between BSO, BSO+ERT, and SM [48]. While past work has investigated the effect of SM on functional connectivity between bilateral hippocampi, no studies have examined the effect of menopause type (surgical or spontaneous) along the longitudinal hippocampal axis. We reasoned that because associative encoding processes may rely more upon anterior than posterior hippocampal function, and bilateral hippocampal connectivity increases across the menopause transition, we might observe important differences [12, 49].

We calculated HRF-weighted correlations between four regions within the left and right anterior and posterior hippocampi across Novel and Repeat conditions. We used mean coordinates for the anterior hippocampus as follows: *X*=28, *Y*=−16, *Z*=−18 for the right hemisphere and *X*=−26, *Y*=−16, *Z*=−18 for the left. The mean posterior coordinates were: *X*=28, *Y*=−30, *Z*=−8 for the right hemisphere and *X*=−26, *Y*=−30, *Z*=−8 for the left [49]. These represent average coordinates showing activation across many cognitive domains, including episodic encoding. Coordinates in the anterior and posterior hippocampus were chosen to provide more specific task-related coverage of the longitudinal axis of the hippocampus.

Through CONN, the BOLD signal in each voxel (from right anterior, right posterior, left anterior, and left posterior hippocampus) was converted to a percent signal change and correlated across the time course associated with each condition (Novel and Repeat). A Pearson correlation was obtained between each region, and a 4×4 correlation matrix was generated for every participant. We used the Fisher *z*-transform to better approximate a normal distribution for the correlations. Using R 4.0.2 (R Core Team, 2019), a linear regression was run on CONN output to remove covariates of no interest (scanner site and history of cancer treatment) across participants. We examined group by condition (Novel and Repeat) interaction effects using PLS, with mean-centering by subtracting condition and group means.

## Results

As expected, BSO, BSO+ERT, and AMC did not differ significantly in age, and SM were significantly older than BSO (*t*(68)=−5.53, *p*<0.0001, Tukey’s HSD test), BSO+ERT (*t*(68)= −6.68, *p*<0.0001, Tukey’s HSD test), and AMC (*t*(68)= −8.51, *p*<0.0001, Tukey’s HSD test). BSO and BSO+ERT did not differ in age of surgical menopause (*t*(44)=1.38, *p*=0.36, Tukey’s HSD test). As expected, with BSO and BSO+ERT entering menopause due to surgery, age of menopause differed significantly, with SM entering menopause at a significantly older age than BSO (*t*(44)= −5.80, *p*<0.0001, Tukey’s HSD test) and BSO+ERT (*t*(44)= −7.30, *p*<0.0001, Tukey’s HSD test). Between BSO and BSO+ERT, there was a significant difference in past cancer treatment history (chemotherapy, radiation therapy, and/or adjuvant therapy), with more women in the BSO group having a history of cancer treatment (*χ*^2^=8.67, *p*=0.01, Chi-squared test). No other demographic measures differed significantly between groups (e.g., APOE4 genotype, depressive mood, and perceived stress measures; Table 1).

On average, women in the BSO+ERT group had initiated their ERT within 1.29 years after their BSO and had been using it for an average of 3.27 years at the time of their scan. For one BSO+ERT participant, PdG level could not be determined; therefore, they were excluded from analyses related to PdG. There was a significant effect of group on PdG level (*χ*^2^=13.52, *p*=0.004): BSO+ERT (*Z*=2.63, *p*=0.01) and AMC (*Z*=3.56, *p*=0.001) had significantly higher PdG levels than SM. No other significant group differences in PdG levels were revealed by post hoc contrasts. There was also the expected significant effect of group on urinary Winsorized E1G level (*χ*^2^=17.05, *p*=0.001): BSO and SM levels did not differ, and AMC and BSO+ERT levels did not differ. However, BSO (*Z*=−1.92, *p*=0.041) and SM (*Z*=2.91, *p*=0.006) had significantly lower E1G levels than BSO+ERT, while BSO (*Z*=2.55, *p*=0.01) and SM (*Z*=3.65, *p*=0.001) had significantly lower E1G levels than AMC.

### Behavior: Associative Memory After BSO

We first asked whether performance on the face-name association task differed between BSO (Combined), AMC, and SM groups. We found a significant effect of group on performance (*F*(2,68)=5.26, *p*=0.008, partial *η*2=0.13), with AMC (*M*=87.43%, *SE*=2.14) having significantly better performance than SM (*M*=76.56%, *SE*=2.61; *t*(68)=3.16, *p*=0.007, Tukey’s HSD test). BSO (Combined, M=84.79%, SE=1.89) also had significantly better performance than SM (*t*(68)=2.48, *p*=0.04, Tukey’s HSD test).

**Fig. 2:**
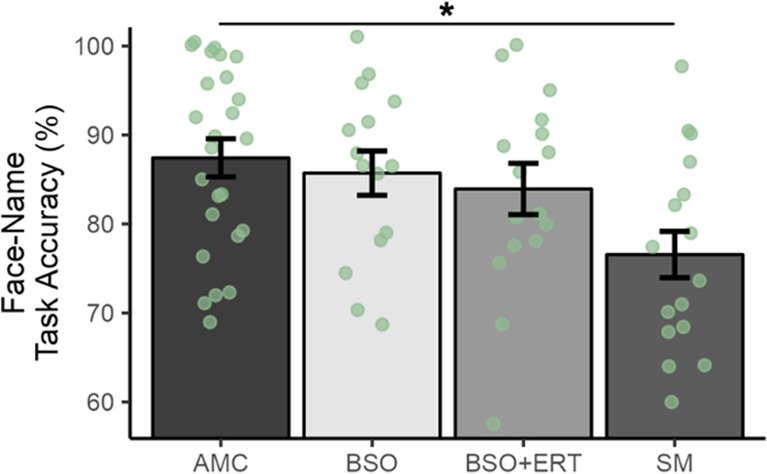
Effect of group on face-name task accuracy (%); age-matched premenopausal control women (AMC) significantly outperformed older spontaneously menopausal (SM) women

When BSO and BSO+ERT groups were separately compared to AMC and SM, there was a significant effect of group on performance (*F*(3,67)=3.55, *p*=0.02, partial *η*2=0.14; Fig. 2). The only significant group difference in task performance revealed by post hoc comparisons was between AMC and SM groups: AMC had significantly better performance than SM (*t*(67)=3.12, *p*=0.01, Tukey’s HSD test). There were no other significant group differences revealed by post hoc comparisons. Thus, BSO did not negatively influence memory performance, although past literature would have suggested that women with BSO and SM would have reduced performance relative to BSO+ERT and AMC.

Women with bilateral salpingo-oophorectomy who were not taking hormone therapy (BSO) and those who were taking 17β-estradiol-based hormone therapy (BSO+ERT) were not significantly different from each other or AMC and SM; error bars represent standard error of the mean; *=*p*<0.05

### Imaging: Regional Brain Function After BSO

We next asked: Is the functional response to encoding of novel face-name pairs in predetermined ROIs different between BSO, AMC, and SM? To determine this, we extracted task-related parameter estimates for Novel–Repeat face-name encoding in some of the same regions in which Sperling and colleagues previously reported less activation for a mixed-sex group of AD patients compared to cognitively healthy elderly adults (Elderly-AD contrast) and less activation for a group of elderly compared to young adults (Young–Elderly contrast), including ROIs in the right posterior medial and lateral hippocampus and left inferior frontal gyrus [8] (ROIs highlighted in Fig. 1).

**Fig. 3:**
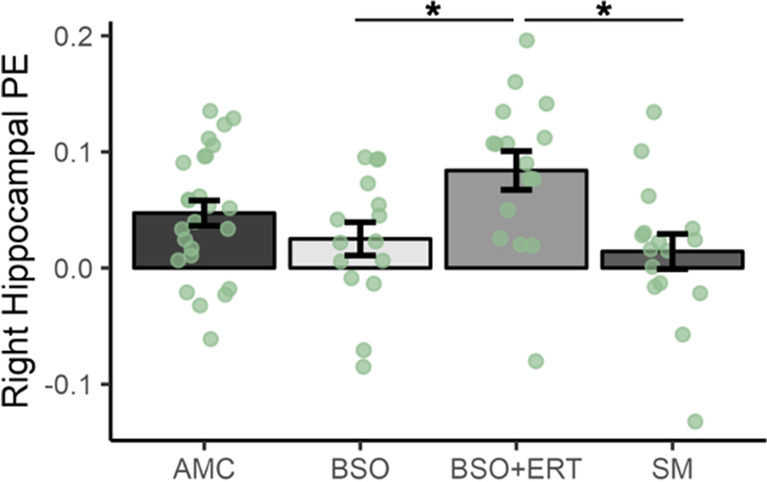
Effect of group on right posterior lateral hippocampal region-of-interest activity; Women with bilateral salpingo-oophorectomy who were not taking hormone therapy (BSO) and women in spontaneous menopause (SM) showed lower activation (mean parameter estimate: PE) than women with BSO who were taking 17β-estradiol-based hormone therapy (BSO+ERT) during Novel compared to Repeat face-name pair encoding; age-matched premenopausal control women (AMC) did not contribute to significant group differences; error bars represent standard error of the mean; *=*p*<0.05

When BSO (Combined) was compared to AMC and SM, there were no significant group differences in average activation for any of the ROIs. However, when BSO and BSO+ERT groups were separated and compared to AMC and SM, there was a significant effect of group on average activation in the right posterior lateral hippocampus (*F*(3,65)=4.07, *p*=0.010, partial *η*2=0.15); both BSO (*t*(65)= −2.84, *p*=0.03, Tukey’s HSD test) and SM (*t*(65)=3.20, *p*=0.01, Tukey’s HSD test) had significantly lower activation than BSO+ERT (Fig. 3). None of the other groups differed significantly in right posterior lateral hippocampus activation according to post hoc contrasts. Given a large effect size of Cohen’s *f*=0.42, a significance threshold of 0.05, 72 participants, and two covariates (scanner site and history of cancer treatment), we determined power for this analysis to be sufficient (power=0.84).

### Correlations

**Fig. 4:**
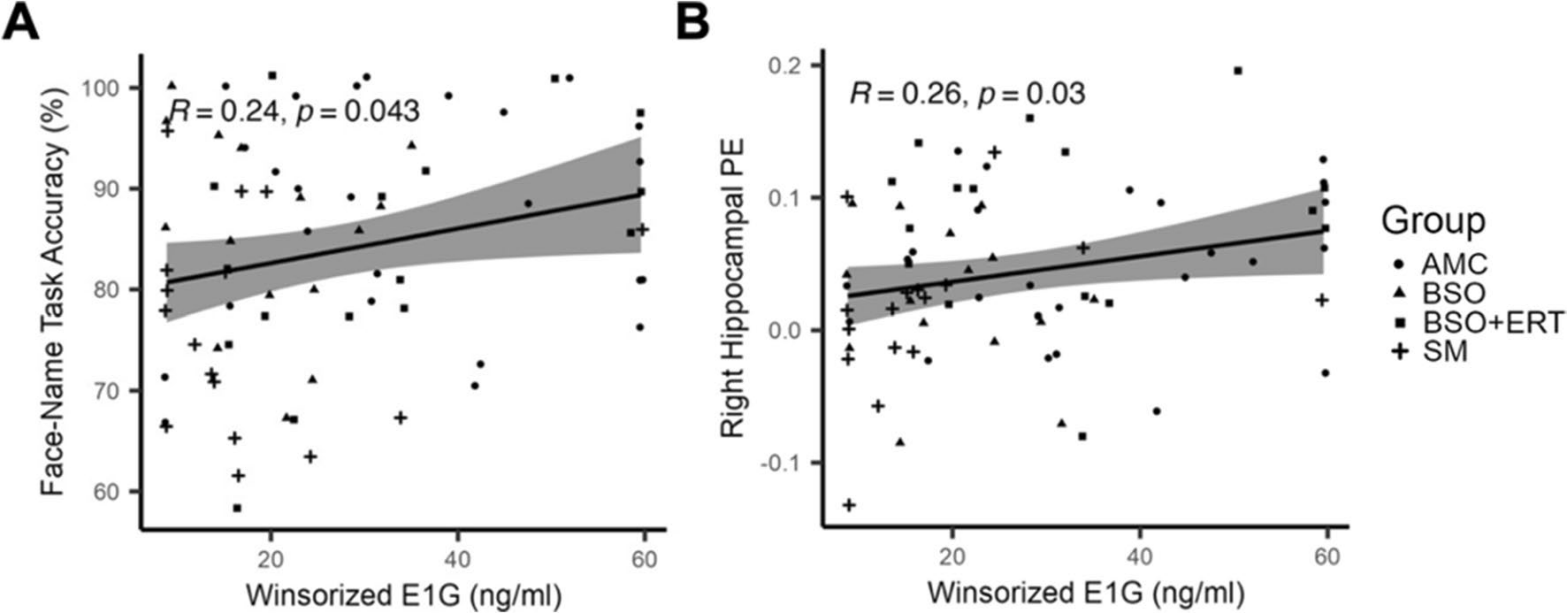
Relationships between urinary ovarian hormone levels, face-name task accuracy, and hippocampal activation; **a)** Plot of correlation between estrone-3-glucuronide (E1G) level and face-name task accuracy; **b)** Plot of correlation between E1G level and right posterior lateral hippocampus activation. **Abbreviations:** SM spontaneous menopause, BSO bilateral salpingo-oophorectomy, BSO+ERT bilateral salpingo-oophorectomy with 17β-estradiol replacement therapy, with or without other hormone therapy types

We sought to explore the potential mechanism underlying significant group differences in face-name task accuracy and right posterior lateral hippocampus activation by correlating these variables with ovarian hormone levels. Across groups, there was not a significant relationship between PdG level and face-name task accuracy (*r*(69)=0.17, *p*=0.16). There was, however, a significant small positive relationship between E1G level and face-name task accuracy (*r*(70)=0.24, *p*=0.043; Fig. 4a).

Across groups, there was not a significant relationship between PdG level and right posterior lateral hippocampus activation, although the small positive relationship was trending toward significance (*r*(69)=0.22, *p*=0.062). There was a significant small positive relationship between E1G level and right posterior lateral hippocampus activation (*r*(70)= 0.26, *p*=0.03; Fig. 4b).

### Imaging: Exploratory Analyses of Whole-Brain Function and Connectivity

**Fig. 5:**
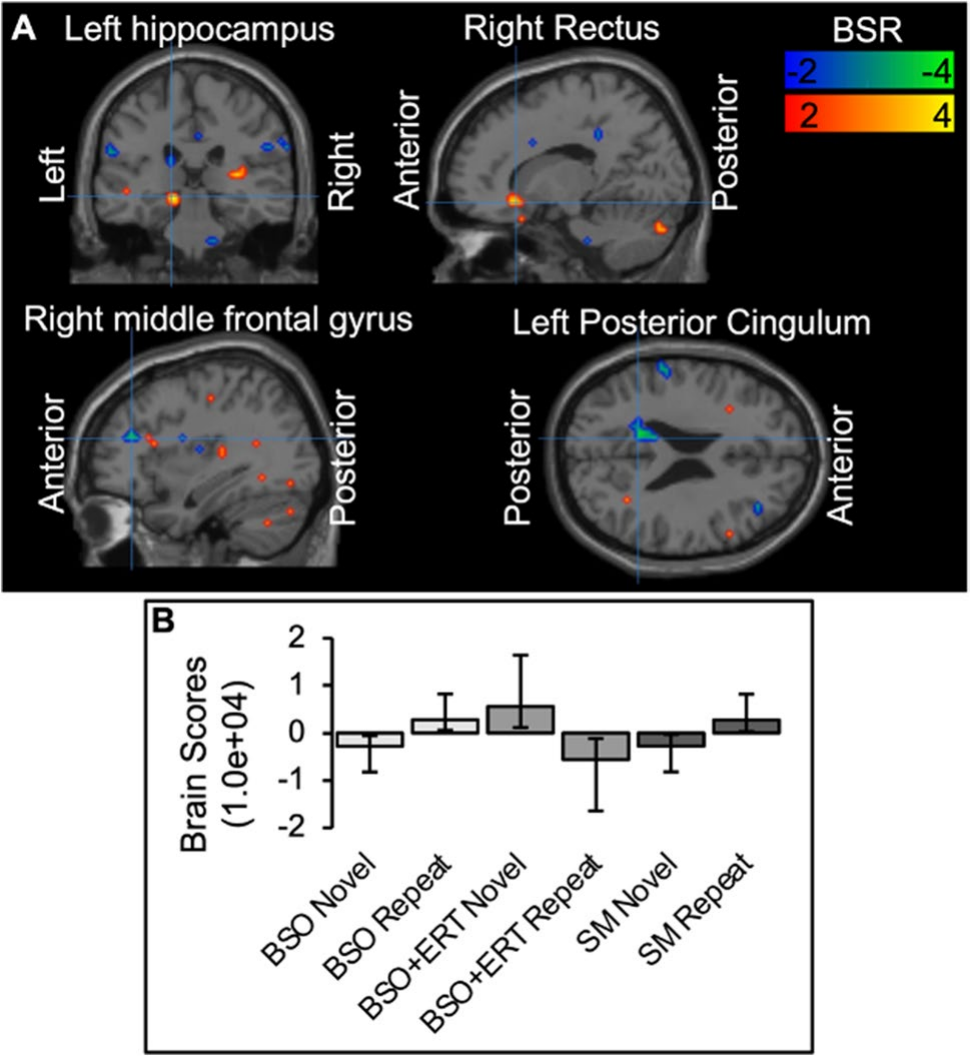
Latent variable 1 (LV1) from task Partial Least Squares: **a)** Spatial maps depict highlighted regions for LV1; *p*=0.008, percent cross-block covariance accounted for 98.25%; **b)** Bootstrap distribution profile for LV1 with 95% confidence intervals; a pattern of Novel and Repeat face-name pair encoding-related activity was significantly different for BSO+ERT compared to BSO and SM; BSO bilateral salpingo-oophorectomy, BSO+ERT bilateral salpingo-oophorectomy with 17β-estradiol replacement therapy, SM spontaneous menopause

Since BSO and SM groups were both experiencing 17β-estradiol loss, we next focused on the differences and similarities between the two different menopause types, BSO and SM, asking whether whole-brain function and hippocampal connectivity after BSO were similar or different from that observed in older women in SM. To assess whole-brain function during Novel and Repeat conditions, we used multivariate PLS. We found one significant latent variable (LV1: *p*=0.008, percent cross-block covariance accounted for 98.25%; Table 2; Fig. 5). The BSO and SM groups showed similar patterns of activity, with increased activity in right middle frontal gyrus and left posterior cingulum—negative salience regions—during encoding of Novel face-name pairs and increased activity in left hippocampus, inferior and middle temporal gyri, inferior frontal gyrus, bilateral rectus gyri, and cerebellum—positive salience regions—during encoding of Repeat pairs. BSO+ERT differed significantly from BSO and SM, showing the opposite pattern, i.e., increased activity in left hippocampus and temporal regions during encoding of Novel face-name pairs and increased activity in frontal and cingulate cortices during encoding of Repeat face-name pairs.

**Table 2:** Local maxima for LV1 identified by task partial least squares

Coordinates (*X*, *Y*, *Z*)	Brain region	BSR	Cluster size (voxels)	*p* value
LV1				
−56, −64, 36	Left angular gyrus	4.80	41	<0.0001
24, −68, −32	Right cerebellum crus 1	4.70	37	<0.0001
−4, 48, −20	Left gyrus rectus	4.63	16	<0.0001
−60, −8, −28	Left inferior temporal gyrus	4.54	41	<0.0001
−60, −36, 4	Left middle temporal gyrus	4.10	39	<0.0001
−12, −64, −48	Left cerebellum 8	4.04	20	0.0001
−48, 36, −16	Left inferior frontal gyrus, orbital	3.88	12	0.0001
40, −16, 60	Right precentral gyrus	3.86	28	0.0001
−24, −68, −20	Left cerebellum 6	3.46	12	0.0005
−16, −28, −8	Left hippocampus	4.72	11	<0.0001
16, 24, −12	Right gyrus rectus	3.81	10	0.0001
20, −84, −32	Right cerebellum crus 2	3.54	19	0.0004
0, 36, −20	Left gyrus rectus	3.52	12	0.0004
−16, −44, 24	Left posterior cingulum	−4.04	13	0.0001
36, 36, 20	Right middle frontal gyrus	−3.60	12	0.0003
−12, −48, 24	Left posterior cingulum	−3.53	14	0.0004

Abbreviations: *LV* latent variable, *BSR* bootstrap ratio

**Fig. 6:**
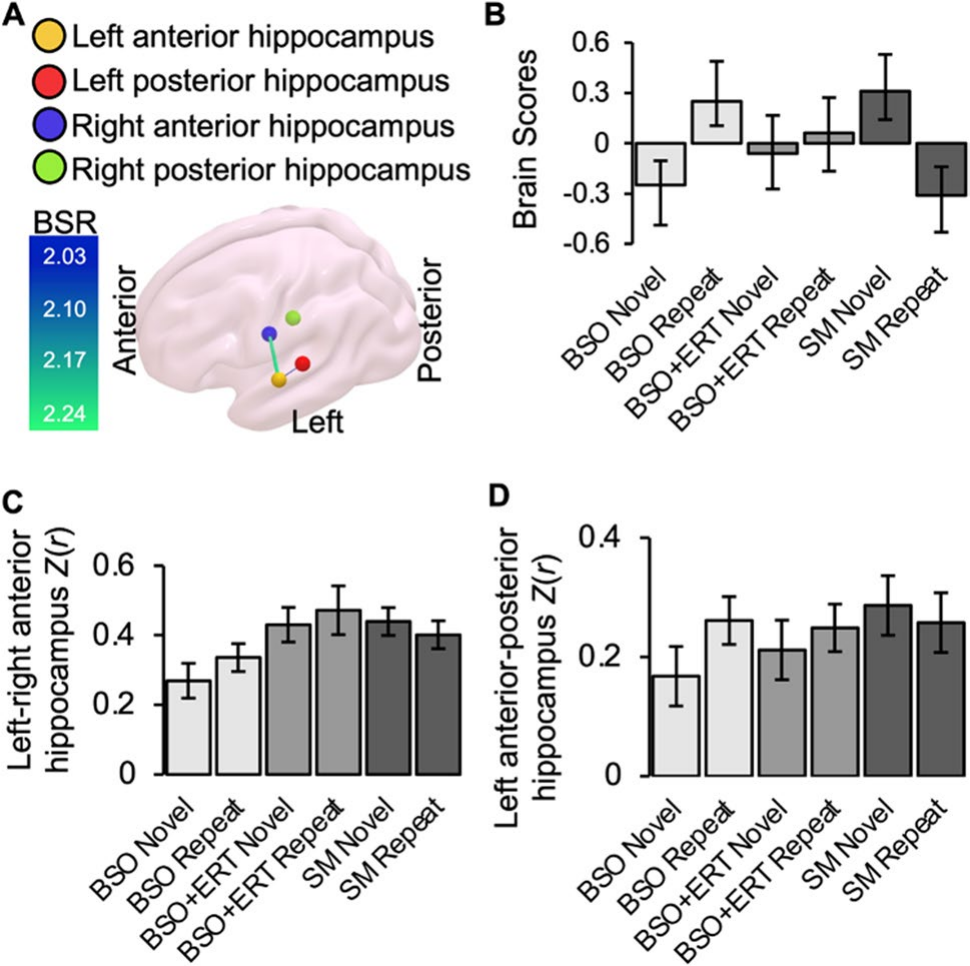
LV1 connectivity partial least squares: **a)** Connectivity between hippocampal regions with significant bootstrap ratios (BSRs) for LV1; *p*=0.07, percent cross-block covariance accounted for 72.91%; **b)** Brain score profile for LV1 with 95% confidence intervals; a pattern of Novel and Repeat face-name pair encoding-related connectivity was significantly different for BSO compared to SM; **c)** Group mean Fisher’s *z*-transformed correlation coefficients between left and right anterior hippocampal ROIs; **d)** Group mean Fisher’s *z*-transformed correlation coefficients between left anterior and posterior hippocampal regions; error bars represent standard error of the mean; BSO bilateral salpingo-oophorectomy, BSO+ERT bilateral salpingo-oophorectomy with 17β-estradiol replacement therapy, SM spontaneous menopause

Using PLS, we next focused on functional connectivity between the left and right anterior and posterior hippocampi of BSO, BSO+ERT, and SM. When we calculated HRF-weighted correlations between four regions within the left and right anterior and posterior hippocampius across Novel and Repeat conditions (Fig. 6a), we found that LV1 explained 72.91% of data variance and trended toward statistical significance (*p*=0.07). Thus, we examined LV1 further to identify differences in hippocampal connectivity. During Novel encoding, compared to Repeat encoding, BSO showed significantly decreased connectivity between left and right and left anterior hippocampi as well as between left anterior and posterior hippocampus (Fig. 6). In contrast, SM showed increased connectivity between left and right anterior hippocampi as well as between left anterior and posterior hippocampus during Novel encoding compared to Repeat encoding. These opposing connectivity patterns suggest that menopause type may influence functional connectivity within the hippocampus during associative encoding. BSO+ERT did not contribute to this latent pattern of effects: i.e., BSO+ERT did not recruit different hippocampal connections between Novel and Repeat conditions.

## Discussion

### Summary of Findings

We explored performance and brain function during an associative memory task in younger women with BSO, comparing them to age-matched premenopausal control women and women in SM. Consistent with past work, AMC outperformed SM on the face-name associative memory task [13]. We did not see behavioral differences between the two BSO groups and the two control groups, which may be because 4–5 years post-BSO is not sufficient to see performance decline. Previous studies have shown that decline in SM is most visible within the first year after menopause and then normalizes, although it is unclear whether women with BSO would follow the same trajectory [50]. Because E1G levels correlated positively with face-name task accuracy, it is also possible that women in the BSO+ERT group either did not have high enough E1G levels or ERT doses were too variable to discern improved performance.

In terms of memory-related brain activity, assessing brain regions reported to decrease in encoding-related BOLD response with age in a mixed sex-cohort, we found that women with BSO had regions of functional brain activity resembling women in SM who were, on average, 10 years older. Both groups were distinct from BSO+ERT. Contrary to past work, AMC did not show associative encoding-related functional brain differences compared to SM. Finally, we found a trend for functional connectivity differences within a hippocampal circuit between BSO and SM, suggesting that type of menopause may also influence hippocampal engagement during associative memory encoding.

To our knowledge, this is the first study of brain activity during an associative memory task in middle-aged women with 17β-estradiol loss and increased risk of AD. Although it is likely that most mixed-sex aging studies of associative memory have included older menopausal women, none have compared the two different types of menopause studied here, midlife BSO and SM. Further, this study is the first to assess hippocampal connectivity in BSO, BSO+ERT, and SM groups, demonstrating that Novel and Repeat face-name pair encoding-related connectivity was significantly different for BSO compared to SM. Thus, in some domains of associative encoding, young women with BSO resemble women in SM, who are 10 years older. However, in other domains, these two groups differ, suggesting that BSO may lead to advanced brain changes with additional features unique to early ovarian removal.

### Women with BSO Show SM-Like Patterns of Neural Activity 10 Years Earlier

In our cohort, without ERT, women with BSO had similar hippocampal, frontal, and temporal cortical activity patterns to SM despite being approximately 10 years younger. Previous work in a mixed-sex cohort demonstrated older adults had decreased hippocampal function compared to younger adults [8]. Importantly, middle-aged women with BSO have decreased hippocampal function. Women in SM have the same results, consistent with work from others showing that without ERT they may have decreased hippocampal function [12, 23]. Further, urinary E1G levels correlated positively with hippocampal activity, consistent with a role for 17β-estradiol in modulating hippocampal function. These findings highlight the role of 17β-estradiol loss, rather than age alone, in affectinginitiating these diverse patterns of brain activity during face-name encoding.

Women with BSO and those in SM also exhibited less repetition-dependent activity reduction in the hippocampus. Notably, this pattern has been observed when comparing mixed-sex mild cognitive impairment groups to cognitively healthy age-matched older adults, suggesting ovarian hormone loss could contribute to these patterns in the aging female brain [51]. Decreased hippocampal activity in women with BSO and SM suggests that BSO may lead to some aspects of an accelerated aging brain phenotype, consistent with other work showing BSO is linked to accelerated aging of multiple bodily systems [52]. We also found, however, that BSO+ERT showed a pattern of decreased hippocampal activation during presentation of repeated face-name pairs, similar to mixed-sex cohorts of younger adults [53]. Thus, ERT may stave off these changes in younger women with BSO.

In women with BSO, multivariate PLS also revealed decreased activation of regions including the inferior frontal gyrus and inferior and middle temporal gyri, and increased activation in the middle frontal gyrus during Novel face-name pair encoding, which may further reflect an early aging pattern. Previously, mixed-sex studies of episodic memory in AD demonstrated hippocampal atrophy associated with reduced temporal function and increased right middle frontal gyrus activation [54]. Again, our results are consistent with the idea that ERT may stave off some of these early brain changes. Further supporting this is the finding that BSO+ERT did not show increased left posterior cingulum activation during novel encoding like BSO and SM.

Interestingly, the posterior cingulum is a region of the default mode network that activates strongly during rest, with suppressed activation during task-based encoding [55]. This deactivation of key default mode network regions, including the posterior cingulum, in tandem with increased hippocampal activation, is necessary for successful associative encoding [56]. Participants with mild cognitive impairment and AD show less deactivation in the posterior cingulate cortex during associative encoding [57], suggesting that, as with older women in SM, women with BSO may have alterations in both activation and deactivation during associative encoding. Thus, patterns typically associated with chronological aging may be partially driven by ovarian hormone loss. Further, these functional posterior cingulate cortex patterns in younger women with BSO suggest that regions other than the hippocampus may be undergoing changes traditionally associated with aging.

### Hippocampal Functional Connectivity Varies by Menopause Type

Despite similar brain activation patterns between BSO and SM, we found group differences in hippocampal connectivity. Functional connectivity analyses revealed that BSO had decreased connectivity between the left and right anterior hippocampius as well as between left anterior and posterior hippocampus during novel face-name encoding. Meanwhile, for SM, connectivity was increased among these regions during novel encoding. Our SM connectivity patterns are consistent with past research showing: (1) in a mixed-sex cohort with advancing age, during resting state, there is increased coupling between the left and right hippocampi [58], and (2) even when controlling for age, women in SM have task-based increases in bilateral hippocampal connectivity compared to men, premenopausal, and perimenopausal women [12].

Thus, comparing menopause types revealed that functional connections supporting face-name pair encoding for women in BSO differed from those of women in SM. This finding further supports the importance of differentiating menopause type and suggests BSO may also lead to different connectivity patterns that might either be compensatory or contribute to higher AD risk.

### Study Strengths and Limitations

Our study focused on a highly under-represented, but nevertheless important group of women for our understanding of the interaction of midlife events, hormones, and aging. To date, this is the first study to investigate the effects of midlife ovarian removal on task-related brain function.

Findings suggest that both early BSO without ERT and SM influence the neural circuitry underlying associative memory. However, our patient cohort, while unique, was small. Still, our cohort size was comparable to others in the field (e.g., [8, 59, 60]), and group comparisons with even fewer participants have demonstrated significant group differences in brain function [60]. Importantly, in smaller cohorts, task-related functional effects are more stable and robust than correlational effects, suggesting our task-related functional group differences would remain regardless of the cohort size [47]. However, it will be important for these findings to be confirmed and extended in larger cohorts.

To date, no known research has investigated the consequences of BSO on brain function. In the absence of large cohorts, “smaller” fMRI studies have found sex differences in brain activity (e.g., with 10 participants per group during long-term memory retrieval [60]). This is particularly relevant for studies of rare populations at increased risk for neurological disorders. These studies, like ours, are likely to have smaller cohort sizes, yet retain the power to identify important group differences [60, 61].

While we did find behavioral differences between AMC and SM groups, AMC did not contribute to any significant group differences in brain function. This may be because AMCs were not all tested at the same point in their menstrual cycles. Given the potential influence of menstrual cycle on the structure and function of specific brain regions involved in associative memory, such as the hippocampus [18, 62], it is possible any effects of 17β-estradiol fluctuation were canceled out in this group due to cycle phase variance. This is an important consideration given that urinary E1G levels were positively correlated with face-name task accuracy and hippocampal function. Future work should focus on menstrual cycle effects on associative memory and its underlying neural correlates.

Many of the significant activation changes observed were between BSO and BSO+ERT. However, while all women taking ERT were taking some form of 17β-estradiol, there was variability in formulation, dose, route of administration, duration, and whether it was administered with progesterone. Importantly, progesterone-based hormone therapy has also been associated with increased hippocampal and prefrontal cortical activation during a visual working memory task in SM [63]. Thus, it would be instructive for future fMRI studies to differentiate between opposed and unopposed ERT as well as between patch and oral administration.

The BSO group also had more cancer treatment history than the other groups; although we statistically controlled for cancer treatment history, we did not have sufficient power to directly examine the effects of cancer treatment on our measures. Importantly, evidence suggests long-term effects of chemotherapy may be limited to only some cognitive domains, with negative effects disappearing after 6 months for domains related to face-name associative memory, such as delayed memory and attention [64].

Additionally, the selected ROI coordinates for univariate analyses came from group differences found in a study using the same task but in a mixed-sex cohort [8]. Previous literature has emphasized that brain activation varies between men and women [12, 60]; thus, our selected ROI coordinates may not be ideal for the women in our cohort. This idea is further supported by our PLS results, demonstrating widespread activation differences between groups beyond the ROIs chosen for univariate analyses. However, it is important to note that the cohort from which we obtained the selected ROI coordinates was primarily female (20/27 participants), increasing the relevance and applicability of these coordinates to our study. We were also unable to take into account the structural variability in the hippocampus: i.e., we were unable to analyze our results by hippocampal subfields that play a key role in associative memory-related brain function [65]. Acknowledging these subfields is critical given rodent studies showing significant effects of 17β-estradiol on dendritic spine density in hippocampal Cornu Ammonis 1 pyramidal neurons and studies of midlife women with early BSO showing hippocampal volume loss is specific to the dentate gyrus and Cornu Ammonis 2/3 composite subfield [26]. Future work should utilize high-resolution fMRI to clarify the precise locations of hippocampal changes, potentially revealing differences in activation between menopause types.

Finally, we found brain activation differences between BSO and BSO+ERT, but no behavioral differences between them. This is not surprising; these are young, highly educated women who have shown other modest but significant changes in verbal episodic and spatial working memory [32] as well as in the volumes of hippocampal subfields [26]. Based on previous brain/behavior studies, we would expect functional brain functional differences to be discernable prior to behavioral changes. Indeed, changes in hippocampal function precede clinical symptoms of mild cognitive impairment progressing toward AD, and divergence in hippocampal function may occur as early as age 40 [66]. Our study underscores the likelihood of this midlife divergence between behavior and brain changes and highlights that even before changes will be apparent behaviorally, hippocampal circuits may function differently due to 17β-estradiol loss and/or aging. Thus, early functional brain changes may be an important biomarker in women with BSO, critical to identifying when to best intervene with treatments or lifestyle changes that might stave off eventual, measurable cognitive decline.

### Conclusions

This is the first study to investigate face-name associative memory-related performance and brain function in younger women with midlife ovarian removal taking and not taking ERT, as well as to compare them to AMC and older women in SM. We found that while BSO+ERT had unique activation patterns during face-name pair encoding compared to BSO and SM, brain activation patterns were similar between women with BSO and SM, suggesting that midlife BSO may bring on brain changes 10 years earlier than they would ordinarily occur. Importantly, we also found potential differences in hippocampal functional connectivity between the two menopause types, suggesting effects of early 17β-estradiol loss could extend beyond an accelerated aging brain phenotype. The changes in memory circuitry related to BSO and its subsequent ovarian hormone loss seem to be evident in early midlife and may precede changes in behavioral performance. Thus, we have demonstrated that the face-name associative memory task we used may be a useful marker for some of the earliest brain changes presaging late-life AD.

## Data Availability

The dataset analyzed during the current study is not publicly available due to restrictions placed by the Research Ethics Board. Our sample is from a small patient population from Canada. Thus, even with de-identified data, participants could be easily identified, and ethics requires we maintain privacy and confidentiality.
